# Organochlorine Pesticides and PCBs in Traditionally and Industrially Smoked Pork Meat Products from Bosnia and Herzegovina

**DOI:** 10.3390/foods9010097

**Published:** 2020-01-17

**Authors:** Brankica Kartalović, Krešimir Mastanjević, Nikolina Novakov, Jelena Vranešević, Dragana Ljubojević Pelić, Leona Puljić, Kristina Habschied

**Affiliations:** 1 Scientific Veterinary Institute Novi Sad, Rumenački put 20, 21000 Novi Sad, Serbia; brankica.kartalovic@gmail.com (B.K.); vet.bmv@hotmail.com (J.V.); ljubojevic.ljubojevicd.dragana@gmail.com (D.L.P.); 2 Faculty of Food technology, Josip Juraj Strossmayer University of Osijek, F. Kuhača 20, 31000 Osijek, Croatia; kristinahabschied@gmail.com; 3 Faculty of Agriculture Novi Sad, Trg Dositeja Obradovića 8, 21000 Novi Sad, Serbia; nikolina@polj.uns.ac.rs; 4 The Faculty of Agriculture and Food Technology (APTF) of the University of Mostar, Biskupa Čule bb, 88000 Mostar, Bosnia and Herzegovina; leonapuljic224@gmail.com

**Keywords:** OCPs, PCBs, smoked pork meat products, pancetta, pork neck, pork tenderloin, sausage, traditional and industrial smoking

## Abstract

The aim of this study was to determine the concentration of 19 organochlorine pesticides (OCPs): (hexachlorocyclohexane (α-HCH, β-HCH, δ–HCH), lindane, aldrin, heptachlor, heptachlor epoxide, *trans*-chlordane, *cis*-chlordane, endosulfane I, endosulfane II, endosulfane sulfate, dichlorodiphenyltrichloroethane (DDT), dichlorodiphenyldichloroethylene (DDE), dieldrin, endrin, dichlorodiphenyldichloroethane (DDD), methoxychlor and endrin ketone and 6 polychlorinated biphenyls (PCBs) (PCB 28, PCB 52, PCB 101, PCB 153, PCB 138 and PCB 180). The samples were taken from pancetta, dry pork neck (budiola), pork tenderloin and sausages produced in Rakitno (Bosnia and Herzegovina), smoked in both a traditional smokehouse and in an industrial chamber. Instrumental analysis was performed using gas chromatography–mass spectrometry (GC–MS). The reliability of the results, i.e., quality control is ensured by standard laboratory practice, which involves participation in proficiency test, the use of blank samples, reference materials and implementation of recommendations given by the relevant international organizations. The concentrations of α-HCH, lindane, PCB 28, PCB 52 and PCB 153 were detected and quantified. The concentrations of OCPs and PCBs did not significantly vary depending on product type and the conditions of production. All the examined samples were for human consumption.

## 1. Introduction

In line with the current trend aimed at revitalizing and supporting traditional food manufacturing processes, autochthonous meat products made from locally available raw materials are becoming more and more significant. Smoking is a traditional method of food preservation and is a quite important in many countries worldwide. Smoked meat products in Bosnia and Herzegovina and the Balkans region alike are made in small smokehouses using open fire. Smoking is used not only to contribute the preservation and extension of shelf life of products, but also to affect sensorial properties of final products [1,2,3,4]. It should be pointed out, however, that the consumption of food produced in a traditional manner carries certain food safety risks. Consumption of foods containing chemical contaminants such as organochlorine pesticides (OCPs) and polychlorinated biphenyls (PCBs) can lead to intoxication, mainly due to long-term exposure [5]. In case of smoked products, the greatest attention is paid to the content of polycyclic aromatic hydrocarbons (PAHs), which are discussed in numerous publications [1,2,6,7,8,9], while little attention is paid to the content of OCPs and PCBs, whose effects on human health are certainly not negligible. PCBs, DDT, HCH gamma isomer (lindane) are potentially carcinogenic, according to the International Agency for Research on Cancer (IARS). IARS listed PCBs in group I, which is made up of the most carcinogenic compound for humans [10]. In addition, very low concentrations of PCBs are known to cause adverse immunotoxicity and neurotoxicity effects in humans [11]. OCPs and PCBs were used in agricultural and industrial activities. OCPs have been extensively used for pest management in agriculture mainly due to their low cost but also high efficiency [5]. PCBs have been widely used as additives in industrial materials including plastics, paints and paper. Moreover, PCBs have been used in electronic industry like transformers and capacitors due to their low electrical conductivity and high thermo-resistance [10]. Because of their adverse effects on human health and environment the usage of most OCPs and PCBs were banned in many countries. However, these compounds are still widely detected both in environment and consequently in food.

It is known that the above-mentioned contaminants are very persistent, characterized by high lipophilic properties, and therefore accumulate in food chain, especially in fats. Consequently, food of animal origin is their main source [12,13]. Many people are most exposed to these compounds (>90%) through the food chain, while inhalation or skin exposure is significantly lower [14,15]. The control of OCPs and PCBs residue in food is important for consumer safety. Different organizations, such as regulatory, advisory and scientific bodies specified different maximum residue levels (MRLs) as well as acceptable maximum intake for these compounds. Numerous analytical methods for the analysis of OCPs and PCBs are widely available. Muir and Sverko [16] highlighted the importance of modern capillary gas chromatography (GC) equipment with either electron capture or low-resolution mass spectrometry (MS) detection for separation and quantification of OCPs and PCBs. There are also different screening methods such as commercially available enzyme-linked immuno-absorbent assays. Different methods for extracting OCPs and PCBs such as liquid:liquid (LLE), cavity-dispersed microwave-assisted (MAE), focused microwave-assisted (FME), solid-phase (SPE), and pressurized fluid (PFE) extraction techniques were previously described [17]. Limits of detection (LODs) and limits of quantification (LOQs) may vary between different methods and between different laboratories.

The aim of this study was the examination of chemical safety of pork meat products smoked in traditional and in industrial conditions. The parameters used to assess chemical safety were OCPs and PCBs. The analyzed PCBs (PCBs 28, 52, 101, 138, 153 and 180) belong to the so-called indicator PCBs that have been proposed to be monitored by several countries and international organizations [18]. The reliability and accuracy of analytical results were achieved by using a GC–MS method that was developed and validated as previously described [19].

## 2. Materials and Methods

### 2.1. Preparation of Samples and Smoking Procedures

All samples of pancetta, dry pork neck (budiola), pork tenderloin and sausages were produced in a local meat industry, which is located in the Municipality of Posušje, Rakitno, Bosnia and Herzegovina. The raw material used for the production of these meat products originated from local meat industry farms. Raw materials were processed using traditional technology. A part of a pig carcass was cut for the production of pancetta in such way that a part of the chest surrounded by the ribs with a big belly was separated from the back by a longitudinal incision and from the neck with cross-section between the third and fourth rib. The bones and cartilage ribs were left as a part of chest. Corresponding part of the belly was separated from the rest of the carcass by transverse section at the level of the lumbal part of spine. Rib bones and cartilage were separated from the chest muscles. The samples of pancetta had a rectangular shape. The samples of budiola were made from previously formed pieces of pork necks. The samples of tenderloin were produced from long dorsal muscle *musculus longissimus dorsi*. After determining the weight of each individual sample, they were salted using a mixture of a mineral and a nitrite salt in a 50:50 ratio. The salting was done manually by using unspecified amount of salt put on the surface of meat in a cooling chamber at the temperature of +4 °C in duration of seven days. The samples were then rinsed with water and moved to a place for drying and smoking where they were dried and tempered for 12 to 20 h. The samples were smoked in both a traditional smokehouse (open combustion chamber) and in an industrial chamber produced by Maurer-Atmos Middleby GmbH., (Reichenau, Germany). In traditional smokehouse, the smoking process for all products lasted 20 days. The traditional products were smoked for 6–8 h per day for first 6 days and after that for 2–3 h every second or third day for 14 days. The industrial products were smoked for 4 h per day for 3 days in the industrial chamber. Experimental conditions during smoking procedures for both traditional and industrial conditions are shown in Table 1. The total time of sample preparation was for 45 days for both methods. Traditional sausages were made using the local recipe, from the mixture of the first-category meat with the addition of 30% of the second-category meat, mineral salt, sweet and hot peppers and minced garlic. The mixture was stuffed in natural casings. Smoking was performed in both a traditional smokehouse and in an industrial chamber. In traditional smokehouse, smoking lasted 20 days and in the industrial chamber for 3 days. The total length of production for industrial and traditional production was 30 days.

### 2.2. Reagents and Materials

All chemicals and reagents used were of analytical grade with high purity. Calibration solutions were prepared using the pesticides mix of 20 pesticides—Organochlorine pesticides mixture produced by Ultra Scientific Inc., (Nort Kingstown, RI, USA), lot CL-1069; PCB Mix 1 that include PCB 28, PCB 52, PCB 101, PCB 153, PCB 138 and PCB 180, produced by Dr Ehrenstorfer, lot G126821IO (Bgm.Schlosser-StraBe 6A-Ausburg, Germany). In order to eliminate the influence of the matrix, calibration through matrix blank sample was performed according to European Commission Document No. SANCO /12571/2013 [20].

**Figure 1:**
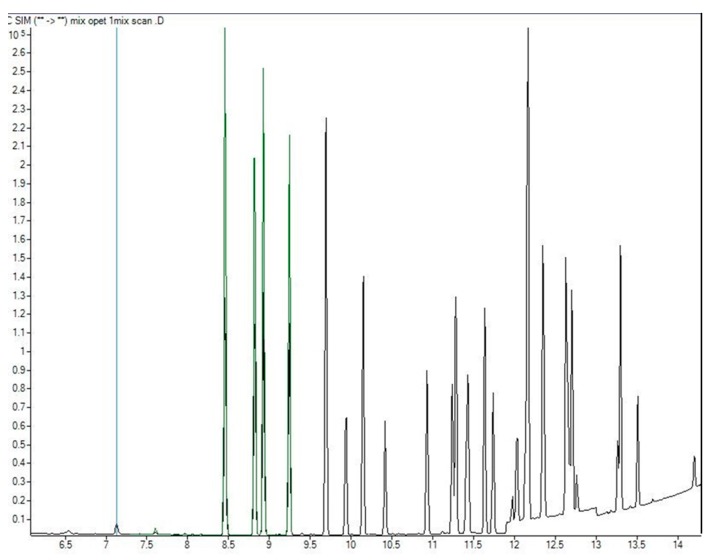
Chromatogram of the standard mixture.

Working standard solution of Pesticides mix that contains 19 pesticides—Chlorinated Pesticides-herbicides lot: 213091108, was obtained from AccuStandard Inc. (New Haven, CT, USA), used for spike preparation for internal control. Spiked samples were used with the purpose of internal control of the following parameters: recovery, precision, limit of quantification (LOQ) and limit of detection (LOD). Chromatogram of the standard mixtures is shown in Figure 1.

### 2.3. Sample Preparation

The method of sample preparation was based on the extraction with acetonitrile (ACN) produced by Sigma-Aldrich (St. Louis, MI, USA) in the presence of anhydrous magnesium sulfate (MgSO_4_) and anhydrous sodium acetate (CH_3_COONa), produced by Merck (Darmstadt, Germany). Sample (3 g) was measured and transferred into centrifuge tube and 3 mL of water and 3 mL of acetonitrile were added. After intensive stirring on a vortex, 3 g of anhydrous magnesium sulfate and 1 g of anhydrous sodium acetate were added. Exothermic reaction occurred within 1 min after the intense stirring on vortex. The sample was then centrifuged for 5 min at 1110× *g* (approximately 3000 rpm). 1 mL of upper acetonitrile extract was transferred into a 5 mL tube, which contained 150 mg of anhydrous magnesium sulfate, 100 mg of primary and secondary amine (PSA) and 50 mg of C18 [21,22,23]. The tube content was centrifuged for 5 min at 1110× *g*. After centrifuging, purified and clear extract was obtained. After that, 0.5 mL of the extract was evaporated in nitrogen and reconstituted with hexane. The sample prepared in this way was ready for the analysis on GCMS-Agilent 7890B/5977A obtained from Agilent Technologies Inc., (Santa Clara, CA, USA).

### 2.4. GCMS Analysis and Instrumentation

The identification of OC pesticides was based on a comparison of retention times of the peaks and target ions with those obtained from a standard mixture of OC pesticides (standards supplied by instrument manufacturer). The quantification was based on external calibration curves prepared from the standard solution of each of the pesticides.

The gas-mass chromatography was Agilent 7890B/5977A MSD (Santa Clara, CA, USA). The GC operating conditions are shown in Table 2. The verification of the peaks was carried out, based on the retention times and target ions compared to those of external OC pesticides. Procedural blank and solvent blanks were analyzed and quantified, but no OC pesticides were found in these blanks. Determination was made in splitless mode, because of that we don’t have split mode, carrier gas was Helium, velocity—32.098 cm/sec; pressure—7.0 psi. Determination was made at constant flow.

### 2.5. Accuracy and Precision

The accuracy of the method was calculated as percent recovery of pesticides from spiked samples. 3 g of homogenized sample was spiked prior to the determination procedure by adding a mixed pesticide standard working solution to reach the final fortification levels of 5, 10, 50, 100 and 500 µg kg^−1^. For each level, five replicates were analyzed. After the addition of each concentration in the matrix, the mixture was equilibrated by shaking, and the samples were allowed to settle for 30 min prior to extraction in order to ensure the sufficient contact of the analytes with the whole matrix. Then, the samples were prepared according to the method which was described earlier.

The precision in case of repeatability (RSDr) was determined at fortification levels of 0.05 mg kg^−1^ with five replicates on the same day. Precision in case of reproducibility (RSDR) was determined at fortification levels of 0.05 mg kg^−1^ with five replicates at three-week intervals. The limit of detection (LOD) was calculated according to Magnusson and Örnemark [24]. In order to determine the LOD of each analyte, ten independent sample blanks fortified at the lowest acceptable concentration of 0.005 mg kg^−1^ were injected, and the LOD was expressed as the analyte concentration corresponding to three times the standard deviation. Limit of quantification (LOQ) was determined according to European Commission Document SANCO/12571/2013 [20]. LOQ was set as the lowest fortification level for each pesticide that was achieved in the acceptable accuracy (mean recoveries for individual pesticides in the range of 70–130%) and precision (RSDr ≤ 20%).

### 2.6. Statistical Analysis

Data analysis was performed using Statistica version 12 from StatSoft^®^ (Tulsa, OK, USA) and Excel (Microsoft Excel, 2007) to determine the descriptive statistic parameters (mean, standard deviation, range) and one-way analysis of variance (ANOVA). ANOVA was used for the assessment of variation in different meat products before smoking and after smoking. Post-hoc Tukey’s test was used for statistical analysis of differences with a statistical significance defined at *p* < 0.05.

## 3. Results

### 3.1. Method Validation

Method validation and quality control were conducted following the European Commission SANTE /11813/2017 [25]. The method was validated in terms of the optimal linearity (*r*^2^ > 0.99). Precision was evaluated by repeatability in triplicate (50.0 µg kg^−1^, *n* = 20) and it ranged from 0.78–17.91%. Recovery ranged from 81.61% to 116.33%. The limits of quantification were lower than the maximum residual limits prescribed for examined products. The obtained results are shown in Table 3 and Table 4.

In calculating measurement uncertainty, the contributions PT (FAPAS: Pesticides and PCB in Milk Powder, July–September 2019, Round 05136), the contribution of reproducibility and contribution of bias were taken into account.

### 3.2. Concentrations of OCPs and PCBs

The study results are presented in Table 5. The concentrations of OCPs and PCBs for each product were examined before smoking, after traditional and industrial smoking and at the end of the manufacturing process. For each product the concentrations of β–HCH, δ-HCH, heptachlor, aldrin, heptachlor epoxide, *trans*-chlordane, *cis*-hlordane, endosulfane I, DDE, dieldrin, endrin, DDD, endosulfan II, DDT, endosulfan sulphate, metoxichlor, endrin keton as well as PCB 101, PCB 138 and PCB 180 were under the limit of detection and limit of quantification in all examined samples. Concentrations of α-HCH, lindane, PCB 28, PCB 52 and PCB 153 were detected and quantified. All these values are below the prescribed maximum residual levels according to European Union legislation [26,27,28,29]. Statistical analysis revealed that there are no statistically significance differences between examined pork meat products before smoking and after smoking and manufacturing.

## 4. Discussion

European Commission (EC) Regulation 178/2006 [26] and European Commission (EC) Regulation 149/2008 [27] prescribed MRLs for DDT and metabolites in various meat products are set at the maximum level of 100 µg/kg. The measured value for DDD was several times below the limits specified in the EU.

The maximum levels for PCBs residues in the EU were set in 2006 [28], allowing maximum levels for sum of dioxins and dioxin-like PCBs expressed in World Health Organization (WHO) Toxic Equivalents (WHO-PCDD/F-PCB-TEQ) for pork and meat products at 1.5 pg/g fat. An amendment of European Commission (EC) 1125/2011 [29], with new limits based on WHO TEF (toxicity equivalency factors) from 2011 set the maximum permitted level of six PCBs at 40 ng/g fat for meat and pork products.

Food and Agriculture Organization (FAO) [30] set the maximum acceptable limits at 500 ng/g DDT and 300 ng/g for β-HCH and lindane. In addition, they determined the maximum total PCBs level at 200 ng/g lipid weight for PCBs congeners 28, 52, 101, 126, 138, 153 and 180. WHO [11] established TDI (tolerable daily intake) for total PCBs at 20 ng/PCBs/kg of body weight per day.

The production and use of PCBs is either banned or restricted in most countries, but these compounds are still very important chemical pollutants found in the environment. They can easily migrate to the food chain, which makes them a significant hazard to human health. The presence of chemical contaminants, including OCPs and PCBs in pork muscle tissue and generally in animal tissues is a direct consequence of environmental pollution [23]. It can also be a result of contaminated pig feed [1]. In the past, the cases of PCBs occurrence in food of animal origin at concentrations higher than regulatory levels were connected with industrially produced animal feed. In recent years, the cases of PCBs level higher than the EU limit most commonly came from either “free range” animals, with the unknown source of contamination [31], or animal feed containing ingredients contaminated with PCBs [32,33]. A case where the source of contamination was determined was an old farm tank stained with PCBs day that peeled off at the place where the pigs were kept [34]. Adequate management can significantly reduce the exposure to PCBs.

The major sources of contamination of food of animal origin with PCBs are the following: PCBs from previously contaminated land, PCBs emitted from buildings and PCBs present on farms [31].

The OCPs and PCBs detected in the examined products at the end of the smoking process were α-HCH, lindane, PCB 28, 52 and 153 while other contaminants were below the detection limit (Table 5). In this study raw material pollutant content was low, but higher than the concentration of the examined contaminants in the products. This is most likely due to free range pig production and smoking process which can affect the concentration of OCPs and PCBs. According to the results obtained by Zabik et al. [35], smoking resulted in significantly higher reductions (40–50%) in OCPs and PCBs levels than other thermal treatments used during culinary processing (baking, cooking). Also, higher concentrations of OCPs and PCBs detected in products after smoking than in the raw material can be explained by higher water content and lower fat content in the pre-smoked samples compared to the post-smoked samples. This was confirmed in the case of PAHs content [1], which at the end of the production process was higher than after the smoking process. Škaljac et al. [36], also found that the concentration of PAHs increases with the length of storage time in traditionally produced sausages.

Some studies have shown higher levels of PCBs than allowed. In the analyzed samples [37], five out of six PCBs indicators were above the recommended maximum limit of 15 µg/kg of fat.

Dioxin-like PCBs (DL-PCBs), including 6 indicator PCBs (PCBs 28, 52, 101, 138, 153, and 180) in meat products analyzed in Germany [38], WHO-PCB-TEQ ranged from 0.06 ng/kg fat for raw ham to 0.13 ng/kg fat for raw sausage. The most common were PCB 118, PCB 126, and PCB 156. In the study conducted in Albania [39], the PCBs profile was the following: PCB 153 > PCB 52 > PCB 138 > PCB 180. The average PCBs content was 6 ng/g. Costabeber et al. [40] examined the concentration of six PCBs in 55 samples of meat (pork and beef) and meat products (different types of sausages, salami, canned meat) from Brazil. They found the following concentrations: PCB 52 (5.18 ng/g fat) > PCB 180 (1.69 ng/g fat) > PCB 101 (1.35 ng/g fat) > PCB 28 (1.19 ng/g fat) > PCB153 (0.47 ng/g fat) > PCB 138 (0.43 ng/g of fat). The sum of 6 congeners was 10.3 ng/g. Meat products have a higher PCBs content than meat (the highest content was in the products made from several types of meat, followed by pork, and the lowest was in cattle). The results of a study conducted by Boada et al. [41] showed that the consumption of meat and sausages increases the risk of DL-PCBs detection in human serum.

OCPs and PCBs belong to the environmental pollutants of anthropogenic origin [42]. Production and use of PCBs is practically completely stopped today, but their remains can still be found primary in electrical installations and the environment, and therefore in food. PCBs have been detected in fish from the Mediterranean 30 years after their ban [43]. DL-PCBs can form during cooking meat. Dong et al. [44] found that the concentrations of DL-PCBs in cooked beef was lower than those in raw beef, but a relatively high concentration of PCBs was detected in the oil vapors generated during meal preparation.

In addition to the concentrations measured in meat and its products, it is also necessary to calculate TDI. TDI for 6PCBs at 10 ng/kg bw (0.01 µg/kg bw) [45,46,47]. The calculation for a 70 kg man is the following: daily intake of Ʃ6PCBs (ng/kgbw) = conc. Ʃ6PCBs (ng/g) × x(g)/70 (kg).

However, most OCPs have not been in use in the EU for many years, but they are still detected in food chain because of their persistence in the environment. Also, the risk can be unconscious and illegal use of OCPs which can lead to food poisoning.

In the present study, α-HCH and lindane were the only OCPs detected under the LOQ values. Lindane (γ-HCH) is the most toxic and the least stable of the HCH compounds. It is transformed into more stable α-HCH over the time [48]. Similar results to these were obtained in a study conducted by Pine and Nuro [39]. They found that average pesticide concentration in meat samples is 13.5 ng/g, while the lowest concentration was measured in pork samples. They also found that the presence of HCHs pesticides was a result previous use of lindane in agriculture.

The report on official controls conducted by Iceland and Norway in 2016 and the results obtained indicate that the potential of EU citizens being exposed to pesticide residues at a concentration that would have a negative impact on health is very low. Products of animal origin (pig fat), were analyzed for the presence of 22 pesticides. 11 of them were below the LOQ, while the remaining 11 were detected and qualified. The most commonly detected and quantified were DDT, hexachlorobenzene and chlordane. The MRL exceeded by a small amount in pig fat samples. For the pesticides for which MRLs are not prescribed, a default MRL of 0.01 mg/kg applies. 11 pesticides were sporadically detected, including DDT, hexachlorobenzene and chlordane in 2.8%, 1.8% and 1%, while the others were present in 0.4% of samples (mainly pesticides whose use is not allowed due to their persistence: dieldrin, α-HCH, lindane). Endosulfane, heptachlor, β-HCH and methoxychlor were not detected in any samples. During 2016, 919 pig fat samples were analyzed, with 97.2% of samples having a pesticide concentration lower than the quantification limit. 2.8% of samples contained one or more pesticides. Up to three different residues were detected and quantified in four samples (0.4%) [49].

As the methodology in analytical chemistry is evolving [50], the number of incidents related to food contamination is expected to increase.

Studies regarding the effect of culinary food processing on OCP concentration showed a significant decrease in OCP levels during thermal processing of food of animal origin [51,52]. Frying, roasting, grilling, cooking and other forms heat treatment of raw meat are effective in reducing pesticide levels in food [53,54]. Concentrations of OCPs and PCBs are associated with the fat content of food. During heat treatment of food, fat is reduced or released from the product, so there is less fat in the final product. Muresan et al. [52] noted that OCPs content decreased only by 1% during cold smoking. After the combined treatment of warm smoke and pasteurization, a reduction of 15% to 16% occurred. Roasting decreased OCPs by 46–56%, respectively. In a review paper [55], it was also concluded that culinary procedures can modify the concentration of contaminants in food. However, this depends on the initial OCPs content in the treated food. Thermal treatments that release or remove fat can reduce the overall concentration of OCPs and PCBs in treated meat and products.

In conclusion, concentrations of α-HCH, lindane, PCB 28, PCB 52 and PCB 153 were detected and quantified. All these values are under the prescribed maximum residual levels. Low levels of contaminants tested could be attributed to increased awareness of pesticides usage and their more proper use, i.e., compliance with the ban on its use, which results in reduction of their presence in the environment. The concentrations of OCPs and PCBs were not significantly affected by product type and by conditions of production. However, further study is required in order to monitor the presence and concentrations of OCPs and PCBs in these products.

## Figures and Tables

**Table 1:** Experimental conditions during smoking procedures of pork meat products.

Sample	Number of Samples	Production Conditions	Smoking Duration (days)	Duration of Production
Pancetta	12	Traditional	20 ^1^	45
Pancetta	12	Industrial	3 ^2^	45
Tenderloin	12	Traditional	20	45
Tenderloin	12	Industrial	3	45
Budiola	12	Traditional	20	45
Budiola	12	Industrial	3	45
Sausage	12	Traditional	20	30
Sausage	12	Industrial	3	30

^1^ first 6 days for 6–8 h per day, 14 days every two or three days for 2–3 h; ^2^ 4 h per day.

**Table 2:** The GC operating conditions.

Descriptions	Conditions
Instrument	Agilent 7890B/5977A MSD (Santa Clara, CA, USA)
Column	Fused silica column (30 m × 0.25 μm film of HP-5M-thickness) Agilent Technologies, Inc., (Santa Clara, CA, USA)
Temperature	Injection 280 °C
	MSD 280 °C
	Column 50 °C (0.4 min hold) to 195 °C at 25 °C/min; hold to 265 °C for 1.5 min at 8 °C/min; maintained at 315 °C for 1.25 min at 20 °C/min
Carrier gas	Helium
Injection volume	4 μL

**Table 3:** The average values of LOD, LOQ, precision, linearity, recovery and RSD in blank smoked meat samples, spiked with 50 µg/kg (*n* = 20).

OCPs	LOD (µg/kg)	LOQ (µg/kg)	Precision (%)	Linearity (*r*^2^)	Recovery (%)	RSD (%)
α-HCH	1.38	4.66	4.26	0.9991	96.13	5.26
β–HCH	0.56	1.89	17.9	0.9992	99.12	8.89
δ–HCH	0.28	1.18	0.78	0.9991	100.3	18.2
Lindane	0.28	1.10	8.88	0.9993	99.50	8.28
Heptachlor	0.27	1.10	3.36	0.9991	88.10	14.6
Aldrin	1.44	4.64	3.62	0.9993	98.31	3.41
Heptachlor epoxide	0.55	1.59	3.49	0.9991	94.39	3.38
*Trans*-chlordane	0.42	1.22	4.42	0.9991	90.22	8.23
*Cis*-chlordane	1.18	3.89	4.28	0.9990	91.56	8.18
Endosulfane I	0.88	2.78	9.26	0.9991	87.27	8.68
DDE	1.38	4.76	3.29	0.9990	96.87	3.88
Dieldrin	1.49	5.20	3.48	0.9992	94.36	3.37
Endrin	0.86	3.12	8.49	0.9998	83.36	16.2
DDD	1.22	3.89	5.72	0.9997	81.61	14.3
Endosulfane II	1.51	4.92	7.81	0.9991	91.21	10.3
DDT	1.38	4.78	3.48	0.9991	94.36	3.37
Endosulfane sulphate	1.45	4.64	13.7	0.9994	116.3	15.3
Metoxichlor	0.59	2.07	7.66	0.9994	106.1	1.78
Endrin ketone	1.26	4.36	5.59	0.9991	85.58	10.5

LOD—Limit of detection; LOQ—Limit of quantification; *r*—Correlation coefficient; RSD—Precision in case of repeatability; DDE—dichlorodiphenyldichloroethylene; DDD—dichlorodiphenyldichloroethane; DDT—dichlorodiphenyltrichloroethane.

**Table 4:** The average values of LOD, LOQ, precision, linearity, recovery and RSD in blank smoked meat samples, spiked with 50 µg/kg (*n* = 20).

PCBs	LOD (µg/kg)	LOQ (µg/kg)	Precision (%)	Linearity (*r*^2^)	Recovery (%)	RSD (%)
PCB 28	0.6	1.9	3.4	0.9994	106.3	3.7
PCB 52	1.0	4.6	7.8	0.9992	101.4	8.6
PCB 101	1.0	3.7	6.4	0.9991	101.1	7.7
PCB 138	1.0	3.2	6.4	0.9990	99.80	7.7
PCB 153	0.9	3.1	5.5	0.9991	110.9	8.3
PCB 180	1.2	4.0	7.3	0.9981	112.3	10

LOD—Limit of detection; LOQ—Limit of quantification; *r*—Correlation coefficient; RSD—Precision in case of repeatability.

**Table 5:** Level of OCPs and PCBs (above the LOD and LOQ values) in different smoked pork meat products (µg/kg).

Sample		α-HCH	Lindane	PCB 28	PCB 52	PCB 153	Ʃ6PCB
Pancetta before smoking	X ± SD	5.1 ± 1.1	15.1 ± 1.08	3.3 ± 0.3	7.003 ± 4.42	3.8 ± 0.1	
	Range frequency	<loq–6.2 33.3%	12.03–28 100%	<loq–10.1 50%	<loq–10.005 33.3%	<loq–4.62 33.3%	<loq–20.1
Pancetta after traditional smoking	X ± SD	<loq	12.5 ± 6.6	<loq	7 ± 6.0005	<loq	
	Range frequency		2.2–26.3 100%		<loq–20 58.3%		<loq–20
Pancetta after industrial smoking	X ± SD	<loq	12.11 ± 1.08	<loq	4.003 ± 2.02	4	
	Range frequency		8.003–14.19 100%		<loq–5 66.7%	<loq–4 16.7%	<loq–8
Pancetta ripening after traditional smoking	X ± SD	<loq	15 ± 14.07	<loq	3.004 ± 4.002	4 ± 1	
	Range frequency		3–46 100%		<loq–9.907 41.7%	<loq–8 83.3%	<loq–15.8
Pancetta ripening after industrial smoking	X ± SD	7.015 ± 5	12.13 ± 5.03	1.2 ± 0.4	1.8 ± 0.2	8.003 ± 6.001	
	Range frequency	<loq–12 33.3%	4.11–22.17 100%	<loq–1.7 83.3%	<loq–6.001 83.33%	<loq–10.005 25%	<loq–10.2
Tenderloin before smoking	X ± SD	30 ± 1	18.15 ± 8.02	<loq	3.001 ± 2.00015	6.005 ± 4.001	
	Range frequency	<loq–34 33.3%	3.12–28.16 100%		<loq–5 66.7%	<loq–10.006 83.3%	<loq–13.5
Tenderloin after traditional smoking	X ± SD	15 ± 4	15.11 ± 2.08	<loq	1.003 ± 0.5	3 ± 1	
	Range frequency	10–20 66.7%	12.01–18.23 100%		<loq–2.004 66.7%	2–6 100%	<loq–6.2
Tenderloin after industrial smoking	X ± SD	<loq	11.16 ± 8.03	<loq	<loq	5.003 ± 6.001	
	Range frequency		3.1–19.19 100%			<loq–10.004 66.7%	<loq–10.004
Tenderloin ripening after traditional smoking	X ± SD	<loq	22.11 ± 7.12	<loq	4.003 ± 2.001	<loq	
	Range frequency		15.004–33.31 100%		1.001–7.005 100%		<loq–7.005
Tenderloin ripening after industrial smoking	X ± SD	<loq	11.17 ± 8.03	3 ± 1	20	<loq	
	Range frequency		10–23 100%	2–4 45.5%	<loq–20 9.1%		<loq–20.4
Dry neck before smoking	X ± SD	9 ± 0.7	17 ± 3	<loq	<loq	3 ± 1	
	Range frequency	<loq–20.02 33.3%	11.11–23.23 100%			<loq–5.005 75%	<loq–5.005
Dry neck after traditional smoking	X ± SD	<loq	11.15 ± 1.14	<loq	3.003 ± 1.004	4.004	
	Range frequency		4.003–31.46 100%		<loq–5.0099 75%	<loq–4.004 8.33	<loq–9.1
Dry neck after industrial smoking	X ± SD	15.05	13.15 ± 3.066	<loq	1.1 ± 0.2	3.007 ± 1.006	
	Range frequency	<loq–15.05 33.3%	11.002–17.26 100%		<loq–1.2 66.7%	2.002–5.02 100%	2.002–6.2
Dry neck ripening after traditional smoking	X ± SD	8.05	15.15 ± 2.1	1 ± 0.4	1.003 ± 0.15	5.007 ± 1.004	
	Range frequency	<loq–8.05 33.3%	12.02–16.28 100%	<loq–2 66.7%	<loq–1.05 66.7%	4.002–6.01 100%	4.002–8.01
Dry neck ripening after industrial smoking	X ± SD	<loq	15.02 ± 2.01	<loq	1.004 ± 0.101	4.1 ± 0.004	
	Range frequency		12.008–16.04 100%		<loq–1.005 66.7%	<loq–4.2 16.7%	<loq–5.2
Sausages before smoking	X ± SD	7.007 ± 5.005	18.12 ± 4.05	<loq	1.18 ± 0.2	1.08 ± 0.6	
	Range frequency	<loq–12.012 33.3%	14.04–22.22 100%		<loq–2.06 83.3%	<loq–2.01 25%	<loq–4.1
Sausages after traditional smoking	X ± SD	<loq	16.16 ± 3.03	<loq	3.003 ± 1.001	2.02 ± 0.01	
	Range frequency		10.1–20.2 100%		<loq–4.004 40%	1.01–3.03 50%	<loq–7.1
Sausages after industrial smoking	X ± SD	<loq	15.25 ± 6.06	<loq	5 ± 2	3 ± 0.4	
	Range frequency		8.20–24.31 100%		2–7 100%	<loq–3.3 25%	2–6.8
Sausages ripening after traditional smoking	X ± SD	<loq	25.27 ± 6.05	<loq	<loq	<loq	<loq
	Range frequency		20.21–31.31 100%				
Sausages ripening after industrial smoking	X ± SD	<loq	27.15 ± 5.06	<loq	<loq	<loq	<loq
	Range frequency		21.08–31.24 100%				

X ± SD—mean ± standard deviation; loq—limit of quantification.
